# Wheat Leaf Rust Fungus Effector Protein Pt1641 Is Avirulent to TcLr1

**DOI:** 10.3390/plants13162255

**Published:** 2024-08-14

**Authors:** Jiaying Chang, Johannes Mapuranga, Ruolin Li, Yingdan Zhang, Jie Shi, Hongfei Yan, Wenxiang Yang

**Affiliations:** 1 Technological Innovation Center for Biological Control of Plant Diseases and Insect Pests of Hebei Province, College of Plant Protection, Hebei Agricultural University, Baoding 071000, China; changjiaying@163.com (J.C.); jmapuranga@hotmail.com (J.M.); m15369870831@163.com (R.L.); 15733719705@163.com (Y.Z.); 2 International Science and Technology Joint Research Center on IPM of Hebei Province, IPM Innovation Center of Hebei Province, Key Laboratory of Integrated Pest Management on Crops in Northern Region of North China, Ministry of Agriculture and Rural Affairs, Plant Protection Institute, Hebei Academy of Agriculture and Forestry Sciences, Baoding 071000, China; shij99@163.com

**Keywords:** *Puccinia triticina*, effector protein, avirulence function, TcLr1, resistance

## Abstract

Wheat leaf rust fungus is an obligate parasitic fungus that can absorb nutrients from its host plant through haustoria and secrete effector proteins into host cells. The effector proteins are crucial factors for pathogenesis as well as targets for host disease resistance protein recognition. Exploring the role of effector proteins in the pathogenic process of *Puccinia triticina Eriks.* (*Pt*) is of great significance for unraveling its pathogenic mechanisms. We previously found that a cysteine-rich effector protein, Pt1641, is highly expressed during the interaction between wheat and *Pt*, but its specific role in pathogenesis remains unclear. Therefore, this study employed techniques such as heterologous expression, qRT-PCR analysis, and host-induced gene silencing (HIGS) to investigate the role of Pt1641 in the pathogenic process of *Pt*. The results indicate that Pt1641 is an effector protein with a secretory function and can inhibit BAX-induced programmed cell death in *Nicotiana benthamiana*. qRT-PCR analyses showed that expression levels of *Pt1641* were different during the interaction between the high-virulence strain THTT and low-virulence strains FGD and Thatcher, respectively. The highest expression level in the low-virulence strain FGD was four times that of the high-virulence strain THTT. The overexpression of *Pt1641* in wheat near-isogenic line TcLr1 induced callose deposition and H_2_O_2_ production on TcLr1. After silencing *Pt1641* in the *Pt* low-virulence strain FGD on wheat near-isogenic line TcLr1, the pathogenic phenotype of *Pt* physiological race FGD on TcLr1 changed from “;” to “3”, indicating that Pt1641 plays a non-toxic function in the pathogenicity of FGD to TcLr1. This study helps to reveal the pathogenic mechanism of wheat leaf rust and provides important guidance for the mining and application of *Pt* avirulent genes.

## 1. Introduction

Plants are attacked by various pathogens during their growth. In the process of interaction between plants and pathogens, on one hand, the pathogens plunder nutrients from the host plant cells to maintain survival and reproduction, while on the other hand, the host plants use various defense mechanisms to inhibit the invasion, growth, and reproduction of pathogens. Pathogens secrete a variety of effector proteins targeting different sites in host plant cells during infection to disrupt a series of defense reactions in plant cells [1,2]. Effector proteins are important pathogen virulence factors, but they are also targets of host disease resistance proteins, making them a double-edged sword [3]. Effector proteins that can be specifically recognized by plant disease resistance (*R*) genes are known as avirulence (*Avr*) genes, while those not recognized are referred to as virulence genes.

Plant disease resistance proteins can recognize effectors through two types of mechanisms, with the most common one being direct recognition, often known as the “receptor-ligand model” [4,5], such as the recognition patterns of wheat stem rust resistance genes *Sr35*, *Sr50*, and *Sr27* with the corresponding *AvrSr35*, *AvrSr50*, and *AvrSr27* of stem rust fungus [6,7,8]. Flax’s L5/L6 proteins directly recognize the pathogen’s effector protein AvrL567 and trigger downstream immune responses [9]. After being transported into plant cells, the rice blast fungus avirulent protein Avr-Pita can directly bind to the LRD region of the N-terminal of the plant Pita protein, triggering downstream signaling pathways to induce disease resistance responses [10,11]. The other type of recognition mechanism is indirect recognition, which comprises two ways. One involves host surveillance proteins recognizing effector proteins and activating disease resistance proteins to trigger immune responses, and it is often referred to as the “guard model”, for example, the recognition mechanisms between RIN4, RPM1, and RPS2 in *Arabidopsis thaliana* with AvrRpm1, AvrB, and AvrRpt2 [12]. The second one involves the effectors targeting decoy proteins and modifying them to elicit disease resistance protein recognition towards the effector proteins, and it is often referred to as the “decoy model” [13,14], for instance, the recognition of RRS1/RPS4 with PopP2 in *Arabidopsis thaliana* [15] and the recognition of soybean Glucanase Inhibitor Protein (GmGIP1) with the pathogen-secreted PsXEG1-Like Protein (PsXLP1) in *Phytophthora soya* [16,17]. These examples clearly illustrate that the recognition mechanisms of disease resistance proteins towards effectors are highly complex. Therefore, gaining a deeper understanding of the interactions between pathogen effector proteins and host disease resistance proteins is crucial for unraveling pathogen pathogenicity and immune induction mechanisms.

Wheat leaf rust is a biotrophic fungus, and the haustorium is its important nutritional organ and the site for secreting effectors [18,19]. However, there are currently few reports about *Pt* avirulent genes. *Pt3* and *Pt27* were identified as candidate avirulent genes corresponding to *Lr9*, *Lr24*, and *Lr26* in wheat [20]. Six hundred thirty-five *Pt* candidate effector proteins were screened through a transcriptome analysis, among which the candidate effector proteins Pt77192, Pt5974, Pt34354, Pt23713, Pt1625, and Pt36553 were upregulated during the interaction between *Pt* and wheat, and these may potentially play diverse roles in the pathogenic process of *Pt* [21]. The integration of the genomic and association analysis approach identified 20 candidate effector proteins for Lr20 [22]. A study based on long-read de novo assembly and comparative genomics also discovered candidate effector proteins for Lr26, Lr2a, and Lr3ka [23]. Recently, transcriptome analysis of a *Pt* mutant strain with virulence against Lr19 identified eight secreted proteins as AvrLr19 candidate proteins [24]. Furthermore, another transcriptome study integrated with computational analysis identified six candidate effector proteins for the leaf rust resistance gene *Lr28*, among which Lr28 was found to strongly bind to the candidate protein c14094_g1_i1, forming a stable complex [25]. Nevertheless, only a few *Pt* effector proteins have been successfully cloned and functionally characterized, such as Pt13024 [26] and Pt_21 [27]. At present, the research on the function of wheat leaf rust fungus effector proteins is still in its early stages. The role of effector proteins secreted by pathogens in pathogenicity still needs to be further studied, and clarification on the function of effector proteins is of great significance for unravelling new methods of disease resistance.

In this study, we screened candidate effector proteins that were highly expressed during the interaction between wheat and *Pt* and obtained a cysteine-rich effector protein, Pt1641, with a secretory function. The effector protein was highly expressed during the initial stages of infection, and its expression trend and level were different in different pathogenic physiological races. Pt1641 targets the cell membrane, inhibits BAX-induced cell death, and triggers H_2_O_2_ accumulation and callose deposition on the wheat near isogenic line TcLr1. Silencing *Pt1641* from the low-virulence race FGD in TcLr1 enhanced the pathogenicity of *Pt* (FGD) on TcLr1, and the infection type changed from “;” to “3”, indicating that Pt1641 is avirulent to TcLr1.

## 2. Results

### 2.1. Pt1641 Is an Effector Protein Secreted by Pt

**Figure 1:**
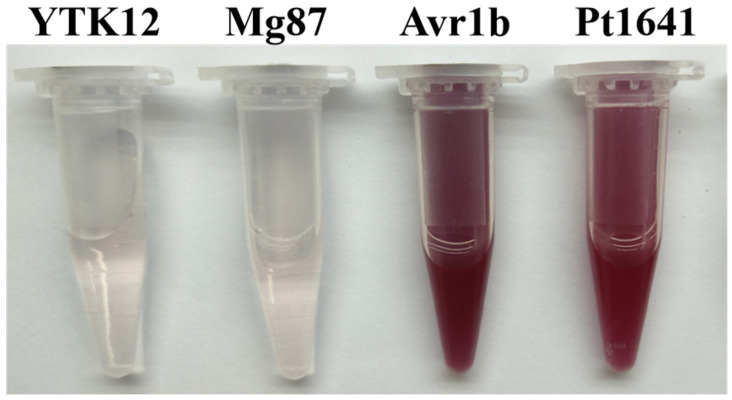
A photograph of microtubes showing the functional validation of the Pt1641 signal peptide using the yeast invertase secretion assay. Yeast strain YTK12 carrying pSUC2-Avr1b served as a positive control, and YTK12 and YTK12 carrying pSUC2-Mg87 were used as a negative control. Secreted invertase can catalyze the reduction of 2,3,5-triphenyltetrazolium chloride (TTC) to form insoluble red 1,3,5-triphenyl formazan (TPF). The presence of a red color confirms the occurrence of invertase activity.

A highly expressed candidate effector protein, Pt1641, was selected from the transcriptome database of *Pt* inoculated on the wheat near-isogenic line Thatcher (Tc). Pt1641 consists of 141 amino acids, including 11 cysteines, accounting for 7.8% of the total, and has a predicted molecular weight of 15.72 kDa. Signal peptide prediction using the SignalP-5.0 online software tool revealed that Pt1641 contains a signal peptide located at amino acids 1–20. ApoplastP predicted that it is localized in the apoplast. Using the TMHMM transmembrane domain prediction online software, we found that the effector protein Pt1641 lacks a transmembrane domain and does not contain any known protein domains. We used the TTC reduction method to detect the enzymatic activity of secreted invertase to verify whether the signal peptide of candidate effector protein Pt1641 has a secretory function. The results showed that the signal peptides of Pt1641 and Avr1b (positive control) can secrete the invertase to hydrolyze sucrose in the solution into monosaccharides. The TTC reaction with monosaccharides formed a red insoluble compound, triphenylmethane (red precipitate), while Mg87 and the empty strain (negative control) could not form a red precipitate, demonstrating that the Pt1641 signal peptide has a secretory function (Figure 1).

### 2.2. Transcriptional Expression Characteristics of Pt1641

**Figure 2:**
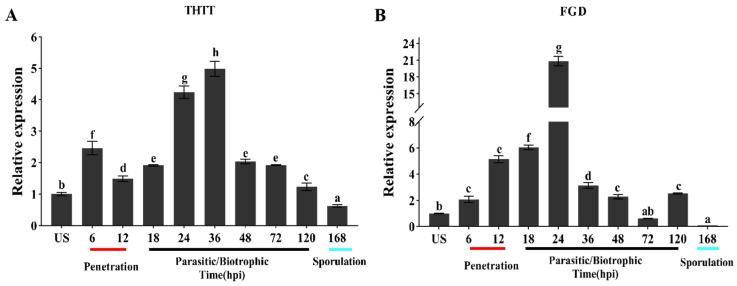
Transcription profiles of *Pt1641* in different *Pt* physiological races. The expression level of *Pt1641* was assayed with RNA isolated from urediniospores (US), and leaves of wheat cultivar Thatcher were inoculated with *Pt* sampled at 6, 12, 18, 24, 36, 48, 72, 120, and 168 hpi. The relative expression was calculated by the comparative 2^−ΔΔCt^ method. The standard deviation and the mean fold changes were calculated with the results from three independent biological replicates. The letters indicate the significant difference compared to urediniospores (*p* < 0.05, unpaired two-tailed Student’s *t*-test). (**A**) Expression patterns of *Pt1641* on Thatcher inoculated with THTT. (**B**) Expression patterns of *Pt1641* on Thatcher inoculated with FGD.

The transcript levels of *Pt1641* were detected by qRT-PCR during the interaction between Tc and two *Pt* physiological races with different virulence. During the interaction between the high-virulence strain THTT and Tc, compared with urediospores, the *Pt1641* expression level was upregulated from 6 h post-inoculation (hpi) and reached its peak at 36 hpi, which is a critical stage for haustoria formation and the suppression of the plant’s initial defense response (Figure 2A). During the interaction between the low-virulence strain FGD and Tc, the expression level of *Pt1641* started to increase at 6 hpi and peaked at 24 hpi, approximately 20 times higher than at the urediospores, a crucial period for the formation of the haustorial mother cells (Figure 2B). At different infection stages, *Pt1641* exhibited higher expression levels in the low-virulence race FGD compared to the high-virulence race THTT, indicating variations in expression levels among different races.

### 2.3. Pt1641 Has a Toxic Function

**Figure 3:**
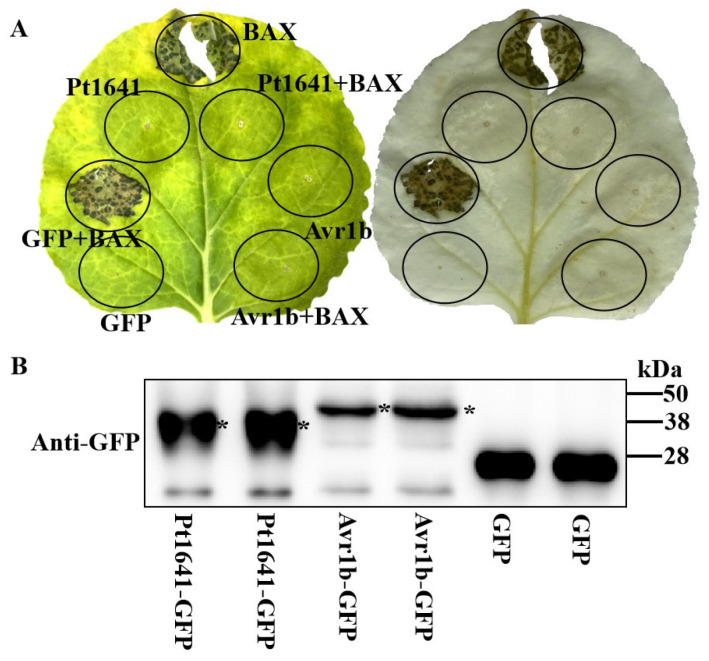
Pt1641 inhibits BAX-induced plant cell PCD. (**A**) Pt1641 was transiently expressed in *Nicotiana benthamiana*, and BAX was injected 24 h later. The same leaf was examined before (**right**) and after (**left**) staining with a decolorizing solution. (**B**) Protein expression was detected by Western blotting. *Agrobacterium tumetericum* containing Pt1641-GFP, Avr1b-GFP, and GFP was injected into tobacco leaves, and BAX was injected 24 h later. After 48 h, proteins were extracted from tobacco leaves and incubated with primary anti-GFP to detect protein expression. * Target protein.

To determine whether Pt1641 possesses potential toxic functions, we tested Pt1641 for its inhibitory effects on BAX-induced PCD in *Nicotiana benthamiana* cells. Both Pt1641 and Avr1b (positive control) inhibited BAX-induced PCD, while the negative control, GFP, could not suppress cell death (Figure 3A). The decolorization of *N. benthamiana* with alcohol resulted in a clear observation of cell death (Figure 3A). WB detection proved that the protein was successfully expressed (Figure 3B). These results showed that the transient expression of effector protein Pt1641 suppressed Bax-induced cell death, indicating that it has toxic functions.

### 2.4. Pt1641 Acts on the Cell Membrane 

**Figure 4:**
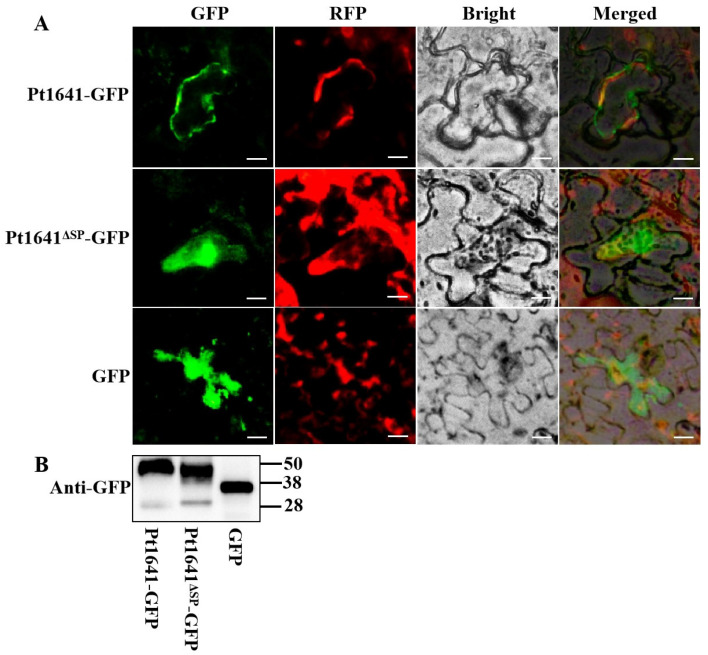
Pt1641 accumulates in the cytomembrane of *N. benthamiana*. (**A**) Leaf tissues of *N. benthamiana* transiently co-expressing Pt1641-GFP, Pt1641^ΔSP^-GFP, and GFP were examined by epifluorescence microscopy. Bars = 50 μm. (**B**) Protein expression was detected by Western blotting. *Agrobacterium tumefaciens* containing Pt1641-GFP, Pt1641^ΔSP^-GFP, and GFP was injected into tobacco leaves. After 48 h, proteins were extracted from tobacco leaves and incubated with primary anti-GFP to detect protein expression.

Identifying the action site of effector protein Pt1641 in plant cells is of great significance for studying its role during the interaction between wheat and *Pt*. Pt1641-GFP, Pt1641^ΔSP^-GFP, and GFP were expressed in *N. benthamiana* cells using a transient expression technique mediated by *Agrobacterium tumefaciens*. The green fluorescence signal was observed in the whole cells of tobacco expressing GFP, and the GFP fluorescence signal was observed in the cell membrane of *N. benthamiana* expressing Pt1641-GFP. In *N. benthamiana* cells expressing Pt1641^ΔSP^-GFP, significant GFP fluorescence signals were observed in the whole cells. These results indicate that the effector protein Pt1641 acts on the cell membrane (Figure 4A). WB detection proved that the protein was successfully expressed (Figure 4B).

### 2.5. Pt1641 Stimulates Callose Deposition and H_2_O_2_ Production on TcLr1

**Figure 5:**
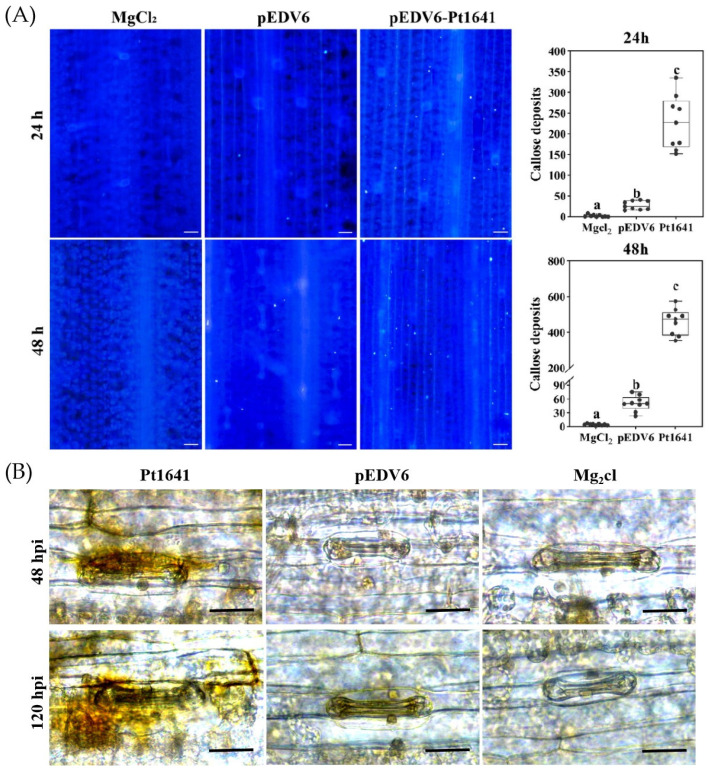
Pt1641 induces callose deposition and the accumulation of H_2_O_2_ on TcLr1. (**A**) Wheat leaf samples were collected at 24 and 48 h after the infiltration of TcLr1 with EtHAn. After decolorization, the leaves were stained overnight with 0.05% aniline blue. MgCl_2_ and pEDV6 served as blank controls. Images were captured under a fluorescence microscope. Bar = 200 µm. The mean values and standard deviations were obtained from nine 1 mm^2^ areas of 3 biological replicates. Letters represent significant differences (*p* < 0.05). (**B**) Wheat leaf samples were collected at 48 and 120 h after the infiltration of TcLr1 with EtHAn. After DAB (1 mg/mL) staining, the accumulation of H_2_O_2_ was observed, and the images were captured under a light microscope. MgCl_2_ and pEDV6 served as blank controls. Bar = 20 µm.

Using the bacterial type III secretion system (T3SS), we overexpressed *Pt1641* in 40 near-isogenic lines (Supplementary Figure S1) and found that it promoted callose deposition and the accumulation of H_2_O_2_ on wheat near-isogenic line TcLr1. Therefore, we selected TcLr1 as the research object. After transiently expressing the effector protein Pt1641 in TcLr1, the effect of Pt1641 on callose deposition and H_2_O_2_ accumulation was observed. The results showed that compared with the leaves injected with EtHAn-pEDV6 and MgCl_2_ buffer, we could clearly observe callose deposition and H_2_O_2_ accumulation in leaves injected with EtHAn-Pt1641, indicating that Pt1641 can induce callose deposition (Figure 5A) and H_2_O_2_ accumulation (Figure 5B) in TcLr1.

### 2.6. Pt1641 Has Non-Toxic Function on TcLr1

**Figure 6:**
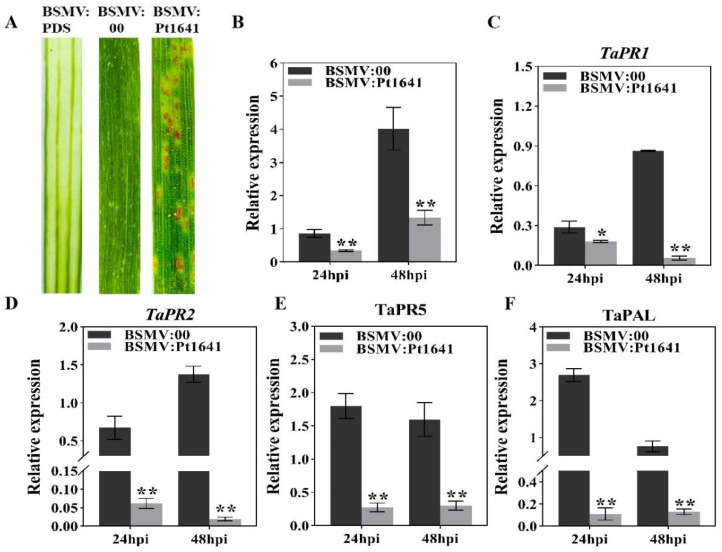
The BSMV-mediated silencing of *Pt1641* in wheat. (**A**) A photograph of phenotypes of wheat leaves of TcLr1 14 days post *Puccinia triticina* inoculation after silencing *Pt1641*. (**B**) The silencing efficiency in *Pt1641* knockdown wheat leaves was assessed using qRT-PCR. The samples were collected for RNA extraction from the 3 leaves of wheat plants at 24 and 48 hpi. The mean and standard deviation were calculated from three independent biological replicates. (**C**–**F**) The expression levels of different genes were assessed in TcLr1. The asterisks indicate a significant difference in samples with *Pt1641*-silenced plants in comparison with the control (* *p* < 0.05; ** *p* < 0.01).

To clarify the function of the effector protein Pt1641 during the infection of the low-virulence physiological race FGD on TcLr1, we employed barley stripe mosaic virus (BSMV)-mediated host-induced gene silencing (HIGS) technology to silence its transcription levels. Plants inoculated with BSMV:*TaPDS* in TcLr1 exhibited bleaching, while mild yellowing and mosaic symptoms were observed on TcLr1 leaves inoculated with BSMV:*Pt1641* and BSMV:00, indicating the effective work of the virus system. The BSMV-inoculated leaves of wheat plants were further challenged with a low-virulence *Pt* race FGD. The disease phenotypes were observed fourteen days post inoculation. Compared with control plants, in TcLr1 plants with silenced *Pt1641*, the pathogenic phenotype of *Pt* physiological race FGD changed from “;” to “3”, and obvious uredinium piles appeared, indicating that Pt1641 plays a non-toxic function in the pathogenicity of FGD to TcLr1 (Figure 6A). qRT-PCR analyses showed that the *Pt1641* expression level was reduced by 59.03–66.79% at 24 and 48 hpi, respectively, in TcLr1 plants inoculated with BSMV:*Pt1641* (Figure 6B) compared to the control plants inoculated with BSMV:00, indicating that *Pt1641* was successfully silenced. 

To clarify the impact of Pt1641 on wheat resistance during the infection process of the *Pt* low-virulence physiological race FGD, we analyzed the expression levels of different disease resistance-related genes using qRT-PCR. The results showed that the expression levels of disease resistance-related genes *TaPR1*, *TaPR2*, and *TaPR5* and salicylic acid synthesis pathway-related gene *TaPAL* significantly decreased in *Pt1641*-silenced plants compared to the control plants (Figure 6C–F), suggesting that Pt1641 can inhibit TcLr1 immune response.

## 3. Discussion

Plants growing in nature face various biological stresses from the surrounding environment. To enhance their survival, plants evolved a more intricate defense system to perceive and defend themselves against plant pathogens [28,29]. However, pathogens can affect the signal transduction and normal function of the host cell by secreting effector proteins that manipulate the host immune responses [1]. Specific disease resistance proteins (R proteins) in plants can directly or indirectly recognize effector proteins secreted by pathogens and trigger plant resistance responses [28]. However, in the long-term co-evolution, new R proteins and effector proteins continue to emerge, thus forming a complex interaction network between plants and pathogens, whereby pathogens endure evolutionary changes to evade host immunity, while hosts are subject to selective pressures that favor the elimination of pathogens [29,30]. Identifying pathogen effector proteins that stimulate *R* gene-mediated disease resistance is of significant practical value in developing disease-resistant plant varieties.

Wheat leaf rust resistance protein Lr1 is a race-specific leaf rust resistance protein and a typical R protein. Since the successful cloning of *Lr1* in 2007, there have been no reports of any effector protein that stimulates the expression of Lr1 protein resistance [31]. Effector proteins are a class of proteins that are synthesized in pathogen cells and then secreted into the extracellular space, then act on the surface of the plant plasma membrane, act in the plasma membrane space, or enter into host cells to exert their effects [1,32]. Therefore, effector proteins are important targets for the recognition of disease resistance proteins in host plants. However, there are few studies on the functional characterization of avirulent genes of wheat leaf rust; for example, PTTG_08198 accelerated the process of cell death and promoted the accumulation of reactive oxygen species (ROS) [33]. Pt13024 was found to inhibit programmed cell death (PCD), trigger ROS accumulation and callose deposition, and exhibit a non-toxic function on TcLr30 [26]. Pt_21 inhibited host defense responses by directly targeting wheat TaTLP1 and suppressing its antifungal activity [27]. Avirulent protein AvrLr15 from *Pt* can induce *Lr15*-dependent immune responses [34]. Hence, the study of the effector protein that stimulates Lr1 resistance is of great significance for the effective control of *Pt* and the development of durable leaf rust-resistant cultivars. In this study, the candidate effector protein Pt1641 was obtained by screening the transcriptome database of wheat infected by *Pt*. A bioinformatics analysis and yeast invertase secretory assay demonstrated that Pt1641 is a secreted protein of *Pt*, which consists of 141 amino acids with 11 cysteine residues and a signal peptide at the N-terminal from the 1–20 aa. 

Pt1641 was overexpressed in 40 near-isogenic lines (Supplementary Figure S1), and its performance varied in different near-isogenic lines. In some near-isogenic lines, it can inhibit the accumulation of callose and H_2_O_2_, while in other near-isogenic lines, it can promote the accumulation of callose and H_2_O_2_ but to varying extents. It significantly promoted callose deposition and H_2_O_2_ accumulation in TcLr1. This implies that Pt1641 was highly recognized by TcLr1 compared to other near isogenic lines. No relevant studies have been reported on TcLr1. In this study, after silencing *Pt1641* in FGD, a *Pt* low-virulence physiological race, its virulence to TcLr1 was significantly enhanced, indicating that Pt1641 plays a non-toxic role in the infection of TcLr1 by FGD. The *Pt1641* expression level and trend in different physiological races were different, and the expression level was relatively high during the interaction between FGD and Tc. Therefore, Pt1641 is easily recognized by Lr1, which may also be the reason why FGD exhibits low virulence on TcLr1. The expression levels of genes in different virulent physiological species have not been studied, so this study provides a new idea for the study of avirulent genes. Currently, most of the studies on avirulent genes have only analyzed their polymorphisms in different physiological species [26,35,36,37], but no specific research has studied the expression of these genes in different physiological races. 

*Magnaporthe oryzae* resistance gene Pita encodes a cytoplasmic membrane receptor protein of 928 amino acids, including NBS and leucine-rich domains, in which amino acid 918 is critical in controlling resistance (resistance is lost when alanine is changed to serine) [10]. The *M. oryzae* effector protein AVR-Pita directly interacts with Pita in a typical mode [10]. On the other hand, Lr1, which belongs to a larger *psr567* gene family, has CC-NBS-LRR domains. A transmembrane domain was predicted at the N-terminal of the Lr1 protein, suggesting that its location may be close to the cell membrane, perhaps as part of a membrane-binding complex [31]. A subcellular localization analysis in *N. benthamiana* revealed that Pt1641 acts on the cell membrane, same as the predicted location of Lr1. An AlphaFold 3 prediction analysis demonstrated a potential interaction between Pt1641 and Lr1, suggesting a high probability of direct interaction [38,39]. Our subsequent experiments aim to unravel and clarify the molecular mechanisms underlying the interaction between Pt1641 and Lr1. Intramolecular disulfide bonds formed by cysteine are thought to play an important role in enhancing the stability of effector proteins in host cells [40]. The effector protein Pt1641 contains 11 cysteine residues, with one cysteine present in the signal peptide. Therefore, in our subsequent research, we aim to further analyze the amino acid sequence polymorphism of Pt1641 in different virulent physiological races as well as the content of its cysteine residues. This analysis will help to elucidate whether the sequence variation in Pt1641 is related to the different defense responses of TcLr1 to different *Pt* virulent races.

This study revealed that the *Pt* effector protein Pt1641 is a secreted protein that functions on the cell membrane, and it can inhibit BAX-induced PCD in *N. benthamiana*. Pt1641 exhibits varying expression levels and trends among different *Pt* physiological races, with relatively higher expression levels during the interaction between FGD and Tc. In wheat near-isogenic line TcLr1, Pt1641 can induce callose deposition and H_2_O_2_ accumulation, indicating that it has the ability to stimulate TcLr1 resistance. Silencing *Pt1641* in FGD on TcLr1 by HIGS resulted in a change in the pathogenic phenotype of FGD from “;” to “3”, indicating enhanced pathogenicity. This suggests that Pt1641 plays a non-toxic role on TcLr1, that is, Pt1641 is avirulent to TcLr1. This study provides a molecular basis for exploring avirulent genes in *Pt*, which can be used to identify corresponding wheat disease resistance genes that can be utilized as tools to carry out genetic engineering research and promote the development of new varieties, which has important practical value for *Pt* disease resistance breeding.

## 4. Materials and Methods

### 4.1. Sequence Analysis 

The Pt1641 sequence was derived from the transcriptome database of wheat infected with *Pt*. Signal peptide identification of Pt1641 was performed using SignalP 5.0 (https://services.healthtech.dtu.dk/services/SignalP-5.0/ (accessed on 1 June 2024)). The localization of the secreted protein was predicted by Apoplast P 1.0 (https://apoplastp.csiro.au/index.html (accessed on 1 June 2024)). TMHMM 2.0 (https://services.healthtech.dtu.dk/services/TMHMM-2.0/ (accessed on 1 June 2024)) was utilized to predict the presence of a transmembrane domain within the effector protein Pt1641. The interaction between Pt1641 and Lr1 was predicted by AlphaFold 3 analysis [38,39]. All primers used in this study are described in Supplementary Data S1.

### 4.2. Expression Analysis of Pt1641

Using Thatcher and Thatcher-Lr1 as the research objects, we inoculated Thatcher with the *Pt* high-virulence strain THTT and the low-virulence strain FGD, and they were maintained at 25 °C. The response of various leaf rust resistance genes to these *Pt* races was explained by Long and Kolmer (1989) [41] in their proposed nomenclature system that designated the virulence of cultures of *Pt* combinations. We collected infected leaves of Thatcher at 0, 6, 12, 18, 24, 36, 48, 72, 120, and 168 h post-inoculation (hpi). Total RNA was extracted from the infected wheat leaves using total RNA purification kit (Sangon Biotech (Shanghai, China)). Subsequently, cDNA was synthesized through reverse transcription using EasyScript One-Step gDNA Removal and cDNA Synthesis SuperMix (Transgen, Beijing, China AE311). qRT-PCR was performed on QuantStudio 5 (Thermofisher, Waltham, MA, USA). The expression level of *Pt1641* was analyzed with *PtActin* as the reference gene. The relative expression of *Pt1641* was calculated by 2^−ΔΔCt^ method. Standard deviations and averages were calculated from results of three independent biological replicates. Statistical significance was assessed using a Student’s *t*-test.

### 4.3. Validation of the Secretory Function of Pt1641 Signal Peptide

To validate the secretory function of the predicted signal peptide of Pt1641, we used the yeast signal sequence trap system [42]. The predicted Pt1641 signal peptide sequence was cloned into the vector pSUC2T7M13ORI (pSUC2) using specific primers, then transformed into the invertase-deficient yeast strain YTK12 and incubated at 30 °C [43]. The positive colonies were screened with CMD-W medium, and then, the invertase enzyme activity was detected through its ability to reduce 2,3, 5-triphenyltetrazonium chloride (TTC) to insoluble red 1,3,5-triphenylformic acid (TPF) [44].

### 4.4. Pt1641 Transient Expression in Nicotiana benthamiana

In order to detect whether Pt1641 can inhibit the PCD induced by Bax, PVX-Pt1641 (without signal peptide) was cloned into pGR106 to construct PVX-Pt1641-GFP plasmid, which was transformed into *Agrobacterium tumefaciens* GV3101. It was then cultured in Luria–Bertani medium containing 25 mg L^−1^ rifampicin and 50 mg L^−1^ kanamycin. GV3101 carrying the corresponding construct was suspended in acetylsyringone buffer (10 mM MgCl_2_, 10 µM AS, 10 mM 2-(N-morpholino) methanesulfonic acid (MES), pH 5.6), and strains containing GFP, Avr1b-GFP, and Pt1641^ΔSP^-GFP with an OD_600_ of 0.5 were injected into 4-week-old tobacco leaves. Twenty-four hours later, the GV3101 strain containing the BAX gene was injected at the same location. Leaf necrosis was observed at 7 days post-infiltration (dpi), and the *N. benthamiana* leaves were decolorized in ethanol/acetic acid (1:1) until semi-translucent, followed by photographing them. To observe the subcellular localization of Pt1641 in tobacco cells, strains containing GFP, Pt1641-GFP, and Pt1641^ΔSP^-GFP were mixed with RFP-labeled GV3101 bacterial solution in a 1:1 ratio and injected into tobacco leaves. After 48 h, TI2-U inverted fluorescence microscope (Nikon, Minato City, Japan) was used to observe and photograph them.

### 4.5. Bacterial T3SS-Mediated Pt1641 Overexpression in Wheat

The constructs pEDV6 and pEDV6-Pt1641 were electroporated into the *Pseudomonas fluorescens* EtHAn. The bacteria were cultured in King’s medium containing antibiotics (50 mg/L spectinomycin and 30 mg/L chloramphenicol) for 48 h. After the bacteria were collected, they were washed twice with 10 mM MgCl_2_, and *Pseudomonas fluorescens* (OD_600_ was 1.0) were infiltrated into the second wheat leaves. Samples were collected at 24 and 48 hpi to detect callose deposition. The leaves were decolorized with acetic acid/ethanol (1:1) for 24 h, then washed twice with 50% ethanol for 15 min each time with a 10 min water rinse, washed with 0.5 M NaOH for 10 min with a 10 min water rinse, and finally washed with 67 mM K_2_HPO_4_ (pH 9.0) for 1 h. Leaf samples were stained overnight with 0.05% aniline blue in 67 mM K_2_HPO_4_ (pH 9.0) [45]. The leaves were rinsed with water, soaked in 50% glycerol, and examined with DAPI filter under TI2-U inverted fluorescence microscope (Nikon). The amount of callose deposition was quantified using ImageJ-win64 software [46]. For the detection of H_2_O_2_ accumulation, the inoculated leaves were sampled at 48 and 120 hpi, and 3, 30-diaminobenzidine (DAB) staining was used to determine the accumulation of H_2_O_2_, which was then observed under a microscope.

### 4.6. BSMV-Mediated Gene Silencing

To investigate the role of Pt1641 during the infection of TcLr1 with low-virulence strain FGD, the *Pt1641* was silenced on TcLr1 by HIGS technique. Specific fragments were cloned and inserted into barley stripe mosaic virus (BSMV) to generate BSMV:γ:Pt1641, which was then transformed into EHA105. Following the protocol used by Qi et al. [44], the BSMV virus was propagated on tobacco leaves. Tobacco leaves containing BSMV were ground in PBS buffer and diatomaceous earth and applied with gloved fingers to the second leaf of two-leaf wheat [47]. BSMV:*TaPDS* encoding a plant phytoene desaturase was used as positive control, and wheat seedlings inoculated with only BSMV:γ were used as negative control. Fourteen days after virus inoculation, the fourth wheat leaves were inoculated with fresh FGD strain and maintained under humid conditions at 25 ± 3 °C. The *Pt* pathogenic phenotype was observed at 14 dpi (infection type: “0” no uredinium or infection court; “;” no uredinium, but there is necrosis or chlorosis; “1” the uredinium are small and have necrotic spots around them; “2” uredinium are small to medium, with necrosis or chlorosis around them; “3” the uredinium is medium, with or without chlorosis; “4” uredinium is large, without chlorosis and necrosis, often satellite uredinium). The leaves inoculated with FGD were collected at 0, 24, and 48 hpi, respectively, for RNA isolation and histological observation. RNA extraction from three leaf samples was performed to evaluate the silencing efficiency. The transcriptional expression of *TaPR1*, *TaPR2*, *TaPR5*, and *TaPAL* was detected using *TaEF* as internal parameter.

## Data Availability

The data presented in the study are available on request from the corresponding author.

## Supplementary Materials

The following supporting information can be downloaded at https://www.mdpi.com/article/10.3390/plants13162255/s1, Figure S1: The effect of P1641 on callose in different near-isogenic lines. The amount of callose on near-isogenic lines was analyzed for significant differences using unpaired two-tailed Student’s *t* test. The asterisk (*) denotes a significant difference in callose deposition between pEDV6 and Pt1641 on the same near-isogenic line (*p* < 0.05). Data S1: Primers used in this study.
