# Supplementary material for: Wheat Leaf Rust Fungus Effector Protein Pt1641 Is Avirulent to TcLr1
Source: Plants (Basel). 2024 Aug 14;13(16):2255. doi: 10.3390/plants13162255 (PMC11359021; doi:10.3390/plants13162255)
Supplement: Supplementary file 1 [file plants-13-02255-s001.zip › plants-3106543-supplementary.pdf]

**Supplementary Data S1. Primers used in this study**

| Primer | sequence(5'-3') | application |
|---|---|---|
| TaPR1-F | GAGAATGCAGACGCCCAAGC | |
| TaPR1-R | CTGGAGCTTGCAGTCGTTGATC | |
| TaPR2-F | AGGATGTTGCTTCCATGTTTGCCG | |
| TaPR2-R | AAGTAGATGCGCATGCCGTTGATG | |
| TaPR5-F | CAAGCAGTGGTATCAACGCAGAG | |
| TaPR5-R | GTGAAGCCACAGTTGTTCTTGATGTT | |
| TaPAL-F | TTCGATTTGCCACCAAGTC | qRT-PCR |
| TaPAL-R | GTGCCTTGGAAGTTGCCAC | |
| QPt1641F | GGGTGAGACGGACGACACAAATG | |
| QPt1641R | CTGACCTGTGCCCGCATTCTTAC | |
| TaEF-F | TGGTGTCATCAAGCCTGGTATGGT | |
| TaEF-R | ACTCATGGTGCATCTCAACGGACT | |
| PtActinF | ATCCACGAGACCACCTACAACT | |
| PtActinR | CAAGATAGAACCACCAATCCATAC | |
| HPt1641F | AAGGAAGTTTAAAGCCATCCGTGAATCTG | Silencing assay |
| HPt1641R | AACCACCACCACCGTGTCGTCCGTCTCACCCT | |
| PGR1641$^{\Delta sp}$F | GTCAGCACCAGCTAGCATCGATATGAAGGAGGCACCGATCGAGCC | |
| PGR1641R | CGCCCTTGCTCACCATCCCGGGAAATTTGGAGTTGGCTGTTA | Sublocalization |
| PGR1641F | GTCAGCACCAGCTAGCATCGATATGCAAAAGATAGTATCATT | |
| Avr1b-F | GTCAGCACCAGCTAGCATCGATACTGAGTACTCCGACGAAACC | |
| Avr1b-R | CGCCCTTGCTCACCATCCCGGGGCTCTGATACAGGTGAAAG | Supression of |
| BAX-F | GTCAGCACCAGCTAGCATCGATATGGACGGGTCCGGG | PCD |
| BAX-R | CGCCCTTGCTCACCATCCCGGGGCCCATCTTCTTCCAG | |
| ZJY1641F | CACCATGCAAAAGATAGTATCATT | Supression of PTI |
| ZJY1641R | AAATTTGGAGTTGGCTG | |
| SP1641F | CCGGAATTCATGCAAAAGATAGTATCATT | Secretion assay |
| SP1641R | CCGCTCGAGTCCGAGCGCCAAACCATGCA | |

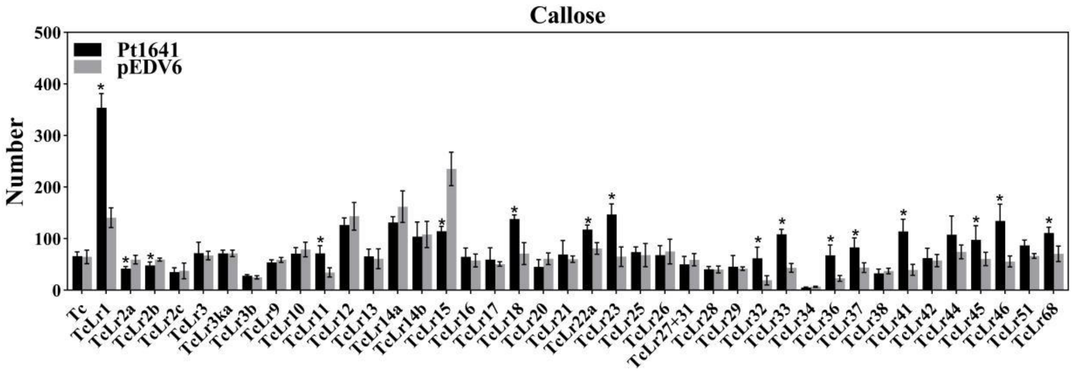


**Supplementary Figure S1**: The effect of P1641 on callose in different near-isogenic lines. The amount of callose on near-isogenic lines was analyzed for significant differences using unpaired two-tailed Student's *t* test. The asterisk (*) denotes a significant difference in callose deposition between pEDV6 and Pt1641 on the same near-isogenic line ($p < 0.05$).
